# Ventilator for the treatment of acute respiratory distress syndrome: A protocol for systematic review and meta-analysis

**DOI:** 10.1097/MD.0000000000013686

**Published:** 2018-12-21

**Authors:** Yan Gao, Ya-long He

**Affiliations:** Department of Neurology, The People's Hospital of Yanan, Yanan, China.

**Keywords:** acute respiratory distress syndrome, efficacy, meta-analysis, safety, systematic review, ventilator

## Abstract

**Background:**

Ventilator has been reported to treat acute respiratory distress syndrome (ARDS). However, its efficacy is still inconclusive. This systematic review and meta-analysis study aims to evaluate its efficacy and safety for the treatment of patients with ARDS.

**Methods:**

The electronic databases of Cochrane central register of controlled trials (CENTRAL), EMBASE, MEDILINE, CINAHL, allied and complementary medicine database (AMED) and 4 Chinese databases will be used to search relevant literature from their inception to the present to evaluate the efficacy and safety of ventilator for ARDS without the language restrictions. This study will only consider randomized controlled trials (RCTs) of ventilator for the treatment of ARDS. The Cochrane risk of bias tool will be utilized to assess the quality of the included RCTs studies. The primary outcomes include arterial blood gases values (recorded once a day) and ventilator settings. The secondary outcomes will include the Acute Physiology and Chronic Health Evaluation II, Simplified Acute Physiology Score, quality of life, cost, death, and any other adverse events. The summary results will be performed by using the models of random-effects or fixed-effects based on the heterogeneity of the included RCTs.

**Results:**

The results will be disseminated to peer-reviewed journals for publication. This study does not need ethics approval, because of no individual data will be involved. The results of this study will help clinicians and health policy-makers to refer for the policy or guideline making.

**Conclusion:**

The results of this systematic review and meta-analysis study may provide helpful evidence for the efficacy and safety of ventilator for ARDS.

**Systematic review registration:**

PROSPERO CRD42018 115409.

## 1 Introduction

Acute respiratory distress syndrome (ARDS) is a serious common respiratory disorder that results from the acute onset of hypoxemia and bilateral infiltrates after a trigger.^[1–4]^ It has been estimated that about 5% of mechanically ventilated patients may affect from this condition.^[5,6]^ In addition, more than 200,000 cases have been reported to suffer from ARDS annually.^[7–9]^ Furthermore, its mortality rate varies from 30% to 60% with significant care costs and debilitating lifelong sequelae for patients with ARDS.^[10,11]^

Despite its high prevalence, the treatment of ARDS still suffered from limited efficacy and poorly supported.^[12–16]^ Mechanical ventilator with ongoing supportive care has reported to benefit for patients with ARDS.^[17–21]^ Although it can cause mechanical ventilation-induced lung injury for patients with ARDS, it still greatly reduces their morbidity and mortality.^[17–21]^ However, presently, no systematic review and meta-analysis was conducted to evaluate the efficacy and safety of ventilator for patients with ARDS. Thus, it is very necessary to conduct a systematic review and meta-analysis to evaluate the efficacy and safety of ventilator for ARDS.

## 2 Methods

### 2.1 Objective

This study aims to evaluate the efficacy and safety of ventilator for the treatment of patients with ARDS.

### 2.2 Study registration

This systematic review protocol has been registered with PROSPERO CRD42018115409. It has been designed based on the previously published guideline of the Cochrane handbook for systematic reviews of interventions and the preferred reporting items for systematic reviews and meta-analysis protocol (PRISMA-P) statement guidelines.^[22]^

### 2.3 Inclusion criteria for study selection

#### 2.3.1 Type of studies

This systematic review protocol will only include randomized controlled trials (RCTs) of ventilator for ARDS without any language restrictions. The studies of any other types will be excluded, such as case reports, Non-RCTs, and quasi-RCTs.

#### 2.3.2 Type of participants

Patients of any age with ARDS, regarding males or females will be considered. However, patients with severe congestive heart failure or severe chronic obstructive lung disease will be excluded.

#### 2.3.3 Type of interventions

Intervention of any type of ventilator treatment will be included. However, the combination of ventilator with other therapies will be excluded. Control intervention will be any kinds of medications, no intervention and others except the ventilator treatment.

#### 2.3.4 Type of outcome measurements

The Primary outcome is the arterial blood gases values (recorded once a day) and ventilator settings. The secondary outcomes will include the Acute Physiology and Chronic Health Evaluation II, Simplified Acute Physiology Score, quality of life, cost, death, as well as any other adverse events.

### 2.4 Search methods for the identification of studies

#### 2.4.1 Electronic searches

The databases of Cochrane central register of controlled trials (CENTRAL, present), Embase (1980 to present), MEDLINE (1946 to present), the Cumulative Index to Nursing and Allied Health Literature (CINAHL, 1982 to present), the Allied and Complementary Medicine Database (AMED, 1985 to present), and four Chinese database Chinese biomedical literature database (CBM; 1980 to present), China national knowledge infrastructure (which includes the database China academic journals) (CNKI; 1980 to present), VIP Information (VIP; 1980 to present), and Wanfang Data (WANFANG;1980 to present) will be searched for the related trials. The search strategy of CENTRAL is presented in Table 1. Similar strategies of other databases will also be built and applied.

**Table 1:**
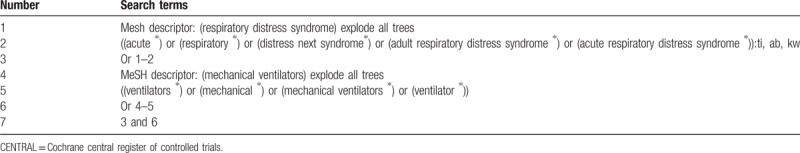
Search strategy applied in CENTRAL database.

#### 2.4.2 Search for other resources

Aside from the electronic databases, the sources of clinical registration will also be searched for ongoing and recently completed studies. Furthermore, the reference list of relevant studies will also be checked to identify additional in order to avoid missing any other eligible studies.

### 2.5 Data collection and analysis

#### 2.5.1 Study selection

**Figure 1:**
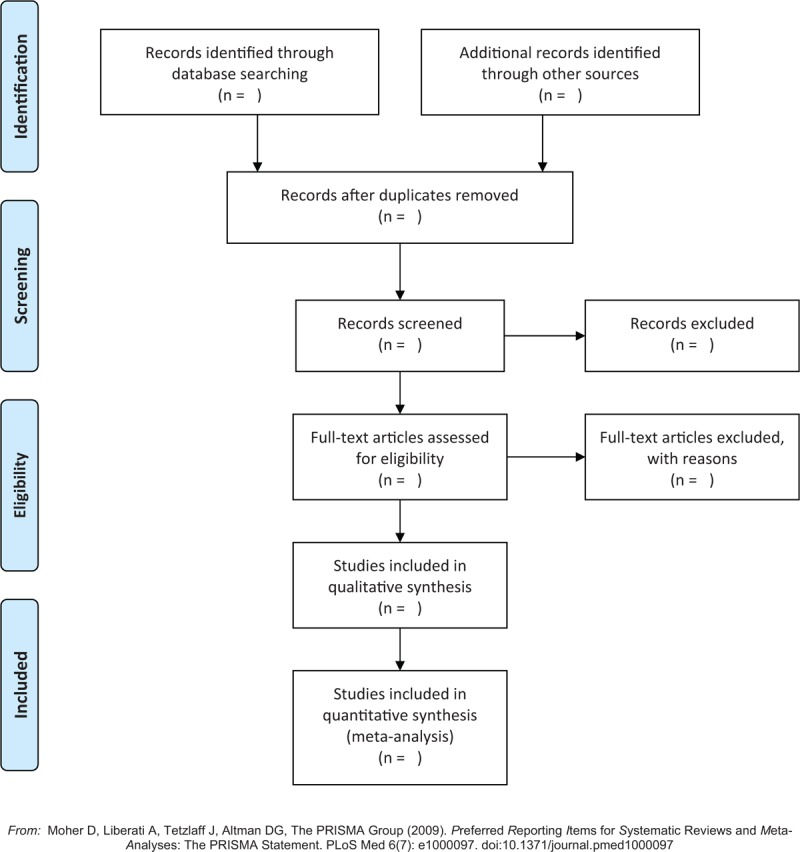
Flow diagram of study selection process.

Two review researchers will independently scan the titles and abstract summary of the potentially eligible studies based on the inclusion criteria and exclusion criteria. If the article fits the inclusion criteria, the full article will be read to further check its eligibility. All the selection procedure will abide by the PRISMA flow chart. If any diverges will occur, a third researcher will solve it by through the discussion. The process of study selection will be presented in Figure 1.

#### 2.5.2 Data collection and management

Two authors will independently extract information from the included RCTs. The information include author, published year, country, disease diagnosis, disease severity, study design, sample size, age, gender, outcome measurements, research results, and adverse events. If the data are missing, wrong, or unclear, the original authors will be contacted to inquire about it. Any other diverge regarding the data collection and management will be resolved by a third reviewer involved through discussion.

#### 2.5.3 Risk of bias assessment of the included RCTs

The Cochrane Handbook for Systematic Reviews of Interventions tool will be used to assess the risk of bias of all included RCTs. This tool includes severe domains covering the random sequence generation, allocation concealment, subjects, investigators and outcome assessor blinding, incomplete results data, selective results reporting, and other bias. Two authors will independently evaluate the quality of each included RCT, and the disagreement will be resolved by discussion with a third author.

#### 2.5.4 Measurement of treatment effect

As for enumeration data, the risk ratio (RR) will be presented. As for continuous data, it will be presented by mean difference (MD) and 95% confidence intervals (CIs). If the measurement tools are not the same, the data will be converted to the standardized mean difference (SMD) and 95% CIs.

#### 2.5.5 Dealing with missing data

If the data are missing, the authors will attempt to contact the authors of original studies to obtain information. If the data are not required, the analysis will be conducted according to the current available data, and it will be discussed as a limitation.

#### 2.5.6 Assessment of heterogeneity

The heterogeneity will be analyzed by the tests of *I*^2^ and *χ*^2^. If *I*^2^ ≤ 50%, the heterogeneity will be ignored, and fixed effect model will be used. *I*^2^ > 50%, significant heterogeneity exist, and random-effect will be applied. If heterogeneity remains significant after the subgroup analysis, a narrative summary will be presented instead of meta-analysis.

#### 2.5.7 Publication biases

Funnel plot will be performed if more than 10 RCTs are included.^[23]^ Furthermore, Egg regression will also be applied for quantitative analysis.^[24]^

#### 2.5.8 Data synthesis

If the heterogeneity is not significant, a meta-analysis will be carried out using RevMan 5.3 software, and a fixed-effect model will be used to pool the data. Otherwise, a random-effect model will be applied, and subgroup analysis or sensitivity analysis will be carried to analyze the reasons that cause the heterogeneity. If the heterogeneity remains significant, a narrative summary will be performed.

#### 2.5.9 Subgroup analysis

Subgroup analysis will be conducted according to the different interventions, controls and outcome measurements.

#### 2.5.10 Sensitivity analysis

Where appropriate, sensitivity analysis will be carried out to eliminate the impact of low-quality studies, and also to evaluate the robustness of the results based on the methodological qualities, and statistical models.

## 3 Discussion

This study protocol of systematic review and meta-analysis will be conducted to assess the efficacy of ventilator treatment to patients with ARDS. To our best knowledge, it is the first systematic review and meta-analysis study to evaluate the efficacy and safety of ventilator for ARDS.

The results of this study will provide a summary of the current evidence on the efficacy and safety of ventilator for ARDS. This evidence will also provide helpful evidence for clinical practice. Moreover, it will also provide evidence for future studies, and health policy-makers.

## Author contributions

**Conceptualization:** Ya-long He, Yan Gao.

**Data curation:** Ya-long He, Yan Gao.

**Formal analysis:** Yan Gao.

**Investigation:** Yan Gao.

**Methodology:** Yan Gao.

**Project administration:** Yan Gao.

**Resources:** Ya-long He, Yan Gao.

**Software:** Yan Gao.

**Supervision:** Ya-long He.

**Validation:** Ya-long He.

**Visualization:** Ya-long He.

**Writing – original draft:** Ya-long He, Yan Gao.

**Writing – review & editing:** Ya-long He, Yan Gao.
